# Investigation on High-Temperature Wear Resistance of Co-Based Superalloys Modified by Chromium–Aluminizing Coatings

**DOI:** 10.3390/ma18061229

**Published:** 2025-03-10

**Authors:** Yang Zhang, Ji Liu, Xuehui Zhang, Jibin Pu

**Affiliations:** 1 School of Materials Science and Engineering, Jiangxi University of Science and Technology, Ganzhou 341000, China; zhangyang1998@nimte.ac.cn; 2 Key Laboratory of Advanced Marine Materials, Ningbo Institute of Materials Technology and Engineering, Chinese Academy of Sciences, Ningbo 315201, China; pujibin@nimte.ac.cn

**Keywords:** GH5188, powder pack cementation, Al-Cr co-aluminization, high-temperature friction and wear, wear mechanism

## Abstract

This study systematically explores and expands upon the research questions, revealing the scientific principles and engineering value of chromium–aluminum (Cr-Al) co-diffusion coatings in enhancing high-temperature friction performance. This study addresses the critical need for wear resistance in GH5188 cobalt-based alloy stator bushings operating in high-temperature environments. The high-temperature wear resistance mechanism of aluminized coatings modified with Cr elements on the GH5188 alloy, based on thermal diffusion technology, was investigated. The experimental results indicate that the high-temperature wear resistance of the samples was directly related to the type and content of oxides in the wear scars and debris. After friction at 700 °C, the aluminized coating on the GH5188 alloy showed the lowest oxide content in the wear scars, primarily composed of CoAl_2_O_4_. The oxides in the wear scars of the GH5188 alloy and Al-Cr co-aluminized coatings were mainly CoCr_2_O_4_ and Cr_2_O_3_, with the Al-Cr co-aluminized coating showing the highest amount of wear debris. The Cr-rich oxide debris not only has high thermodynamic stability but also exhibits relatively low high-temperature growth stress, making it difficult to spall. Additionally, the higher diffusion coefficient of Cr^3+^ accelerates the reoxidation of wear debris pits, resulting in excellent high-temperature wear resistance. The wear rate of the Al-Cr co-aluminized coating was reduced by 30% compared with the GH5188 substrate and by 69% compared with the aluminized coating. In summary, the key findings are not only applicable to cobalt-based alloys but can also be extended to a broader range of material systems and engineering applications. This provides new perspectives and methodologies for the design of high-temperature coatings, the development of materials for extreme conditions, and interdisciplinary applications.

## 1. Introduction

The GH5188 cobalt-based superalloy is widely used in advanced aero engine components such as stator bushings and turbine sealing rings due to its excellent high-temperature strength (>800 °C), superior thermal corrosion resistance, and good thermal fatigue resistance [1]. With the continuous increase in the thrust-to-weight ratio and turbine inlet temperature of aero engines, the operating environment for stator bushings has become increasingly severe. These components must endure long-term exposure to high-temperature gas environments (700–900 °C) while facing centrifugal loads, wear, and high-temperature adhesion [2], leading to losses of dimensional accuracy and potential seizure failures, which severely threaten engine reliability [3]. Although the traditional GH5188 alloy has been optimized for high-temperature mechanical properties through solution strengthening and carbide precipitation, its tribological performance still fails to meet the longevity demands of new-generation engines [4]. Therefore, developing surface functional coatings with high-temperature wear resistance has become a key strategy for enhancing the tribological performance of the GH5188 alloy [5].

Preparing an aluminizing coating using the solid powder embedding method is an important approach to enhance the high-temperature friction performance, surface properties, and reinforcement of cobalt-based superalloys [6]. In recent years, aluminizing coatings on the GH5188 alloy have demonstrated excellent oxidation resistance at 700 °C, with the formation of a continuous α-Al_2_O_3_ film on the surface effectively inhibiting oxygen diffusion into the substrate [7]. Additionally, the aluminized layer can reduce the risk of high-temperature adhesion by decreasing atomic interdiffusion tendencies between friction pairs, a characteristic particularly important in the blade-casing contact pairs of aero engines [8,9]. However, existing studies mainly focus on the oxidation resistance and anti-adhesion properties of aluminizing coatings, with significant gaps in the systematic study of their high-temperature tribological behavior. For example, Chen et al. [10] found that the brittleness of the α-Al_2_O_3_ film in aluminizing coatings leads to early spallation during high-temperature sliding wear, exposing unoxidized metal surfaces and inducing abrasive wear. Zhang et al. [11] further pointed out that the hardness mismatch between the aluminized layer and nickel-based counterpart exacerbates interfacial plastic deformation and material transfer, increasing the wear rate to 2–3 times that of the substrate alloy. Critically, the tribological performance of aluminizing coatings is closely related to their microstructure, yet there is a lack of in-depth exploration of the relationships among process parameters, coating structure, and wear mechanisms in the literature [12]. These limitations indicate that a single aluminizing coating cannot achieve the synergistic optimization of anti-adhesion and wear resistance properties, necessitating breakthroughs through multi-alloy modification to overcome performance bottlenecks.

To overcome the performance limitations of single aluminizing coatings, researchers have proposed the introduction of Cr elements to construct Al-Cr co-deposited coatings. This approach aims to achieve a synergistic enhancement of anti-adhesion and wear resistance properties [13]. Theoretical analysis and experimental studies indicate that the introduction of Cr improves coating performance through the following mechanisms. The (Al, Cr)_2_O_3_ composite oxide film, formed by Cr_2_O_3_ and Al_2_O_3_, exhibits higher density and self-healing capabilities, significantly delaying the rupture of the oxide film at high temperatures [14,15,16]. Solid solution strengthening by Cr and nano-scale Cr-rich precipitates increase the coating hardness (HV ≥ 600), enhancing its resistance to plastic deformation and abrasive wear [17,18,19]. Additionally, the addition of Cr can adjust the thermal expansion coefficient compatibility between the coating and substrate (Δα reduced by 15–20%), reducing interfacial stress concentration during high-temperature friction processes and thus inhibiting coating spallation [20].

Current research on the introduction of chromium (Cr) to form Cr-Al co-diffusion coatings primarily focuses on nickel-based alloys to achieve a synergistic improvement in anti-adhesion and wear resistance. However, systematic studies on Cr-Al co-diffusion coatings for GH5188 cobalt-based alloys have been overlooked, and the influence of Cr-Al coatings on high-temperature friction interfaces, such as the competition between oxidation and wear mechanisms, remains unclear [21,22,23,24]. This work specifically addresses the challenges of controlling high-temperature wear mechanisms by investigating the evolution of oxide compositions and valence states at the friction interface, achieved through the preparation of Cr-Al co-diffusion coatings via pack cementation. In terms of tribological behavior, studies by Su et al. [25] on nickel-based alloys have shown that Al-Cr co-deposited coatings can reduce high-temperature wear rates by more than 40% compared with single aluminizing coatings. This reduction is attributed to the enhanced toughness of the oxide film and the synergistic lubricating effect of friction–chemical reaction films (such as CrO_3_-Al_2_O_3_ mixed layers) promoted by Cr [26,27]. However, these studies have primarily focused on nickel-based alloy systems. Systematic research on Al-Cr co-deposited coatings for GH5188 cobalt-based alloys remains extremely limited [28]. Currently, the mechanisms by which Cr-modified aluminizing layers on cobalt-based alloys affect high-temperature frictional interface reactions (such as the oxidation-wear competition mechanism) are not yet clear [29].

In summary, this work focuses on the study of the friction and wear behaviors and mechanisms of the GH5188 alloy and its aluminized and Al-Cr co-deposited coatings at 700 °C. Aluminized and Al-Cr co-deposited coatings were prepared on the surface of the GH5188 alloy using the solid powder embedding method. The tribological performance of the GH5188 alloy substrate, aluminized coating, and Al-Cr co-deposited coating was evaluated using a high-temperature friction and wear system. The laser scanning confocal microscopy (LSCM), field emission scanning electron microscopy (FESEM), X-ray photoelectron spectroscopy (XPS), and X-ray diffraction (XRD) techniques were used to elucidate the composition of oxidation products, evolution of chemical valence states at the friction interface, and their role in wear mechanisms. This study provides scientific evidence for the composition design and process optimization of protective surface coatings for high-temperature components in aero engines.

## 2. Experimental Materials and Methods

### 2.1. Experimental Materials

In this study, the commercially available GH5188 (Zhejiang China) cobalt-based high-temperature alloy was selected as the research subject. The primary elements and their contents are detailed in Table 1.

In this study, the GH5188 alloy was used as the substrate, with sample dimensions of 20 mm × 20 mm × 3 mm. Aluminized and Al-Cr co-deposited coatings were prepared on the GH5188 alloy surface using the solid powder embedding method. The aluminizing agent components included aluminum powder as the aluminum source, ammonium chloride as the activator, and alumina as the filler. For the Al-Cr co-deposition, aluminum powder served as the aluminum source, chromium oxide as the chromium source, ammonium chloride as the activator, and alumina as the filler. Different heat diffusion parameters led to varying coating thicknesses without altering the phase structure. Therefore, theoretically, these parameters should not have significantly affected the high-temperature friction performance of the coatings [30]. In this study, we discuss the friction performance of coatings prepared with specific co-diffusion parameters. The specific compositions of the embedding agents and the parameters for the thermal diffusion experiments are detailed in Table 2.

### 2.2. Preparation of Coatings

After degreasing and cleaning with deionized water, the GH5188 alloy samples were sanded and polished using sandpaper up to 2000 grit. The surfaces were then ultrasonically cleaned in acetone for 10 min, rinsed with ethanol, and air-dried. The samples and prepared embedding agents were placed in a crucible, ensuring no contact between the samples and the crucible walls, as well as that the embedding agents completely covered the samples. The crucible was then sealed. Thermal diffusion experiments were conducted according to the parameters specified in Table 2 to prepare the aluminized and Al-Cr co-deposited coatings. After the experiments, the samples were furnace-cooled to room temperature. Throughout the process, argon gas was used as a protective atmosphere to prevent sample oxidation.

### 2.3. High-Temperature Friction Experiments

High-temperature friction experiments were conducted on the GH5188 alloy, aluminized coating, and Al-Cr coating samples in an air environment at 700 °C. The equipment used was a high-temperature friction and wear tester (THT 1000, Anton Paar, Graz, Austria). The experimental conditions were a load of 5 N, a friction radius of 3 mm, a friction speed of 10 mm/s, and a counter body of a 6 mm Si_3_N_4_ ball. After the friction tests, the wear scar profile dimensions were measured multiple times using laser confocal microscopy (VK-X1000, Keyence, Osaka, Japan). The average value of these measurements was used to determine the wear scar cross-sectional area S. The wear rate under different high-temperature friction conditions was then calculated using the following formula:

(1) w=(S×2πr)/F×L

where S represents the wear scar cross-sectional area (μm^2^), r is the friction radius (mm), F is the applied load during friction (N), and L is the total friction distance (m).

### 2.4. Characterization and Performance Evaluation of Coating Structures

The microstructure and surface wear scar characteristics of the GH5188 alloy, aluminized coating, and Al-Cr co-diffusion coating before and after high-temperature friction were characterized using a thermal field scanning electron microscope (ZEISS-300, Baden-Württem, Germany) with an accelerating voltage of 15 kV. Elemental distribution in the diffusion layers and wear scar surfaces was analyzed using an energy dispersive spectrometer (EDS). The surface hardness and cross-sectional hardness gradients of different diffusion layers were measured using a Vickers hardness tester (VH-3000, Wilson, New York, NY, USA) with an indenter load of HV_0.2_. Each set of data was tested in triplicate, and the mean values and standard deviations were calculated. The phase analysis of the GH5188 alloy and diffusion layers was conducted using an X-ray diffractometer (ADVANCE D8, Keens, Tokyo, Japan) with a scanning range of 20° to 90° and a scan rate of 0.05°/s. The oxide structures of non-wear scar areas after high-temperature friction tests were analyzed and characterized using a two-dimensional small-angle X-ray scattering instrument (SAXS, Xeuss 3.0 UHR, XENOCS SAS, Paris, France) with an incident angle of 0.5°, a scanning range of 0° to 60°, and a scan rate of 0.05°/s. X-ray photoelectron spectroscopy (AXIS SUPRA+, Shimadzu, Tokyo, Japan) was employed to analyze the chemical states of oxides on the wear scar surfaces of the GH5188 alloy and the various coating samples after high-temperature friction tests, providing a more detailed understanding of the oxidation characteristics.

## 3. Results and Analysis

### 3.1. Cross-Sectional Morphology and Surface Phase Analysis of Coatings

**Figure 1:**
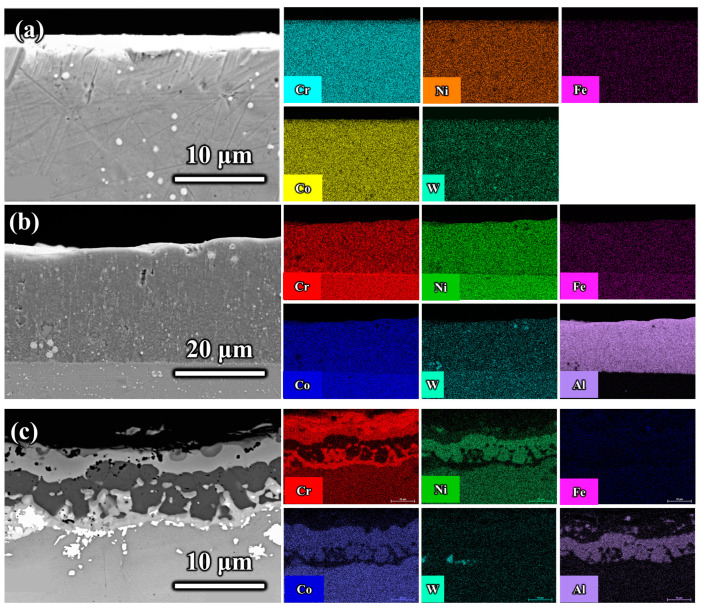
Cross-sectional morphology and element distribution of different samples: (**a**) GH5188 alloy; (**b**) aluminum-diffusion coating; (**c**) Al-Cr co-diffusion coating.

**Figure 2:**
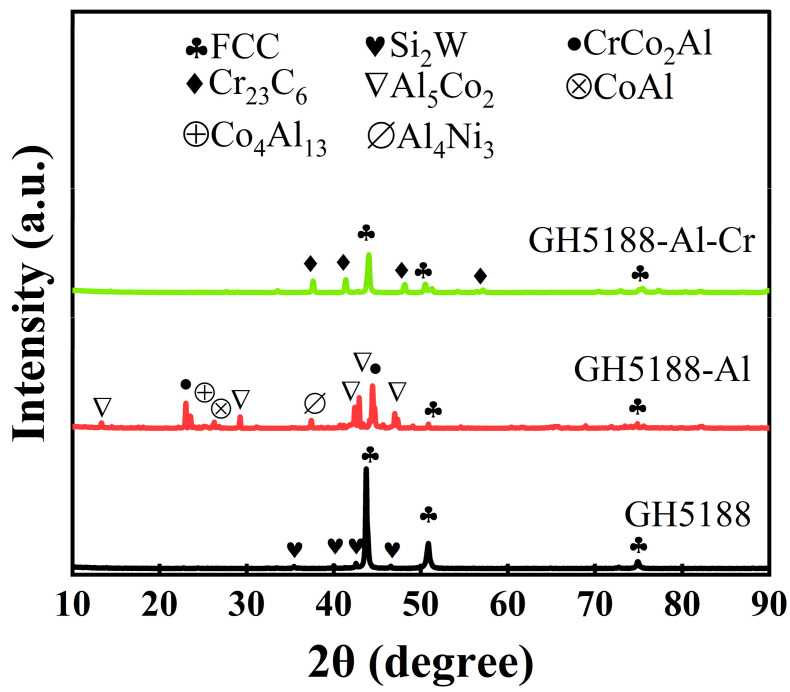
X-ray diffraction patterns of the GH5188 alloy, aluminum-diffusion coating, and Al-Cr co-diffusion coating before high-temperature friction.

The microstructure and elemental composition of materials are crucial factors affecting their friction performance. Therefore, this section examines the microstructure and elemental distribution of the GH5188 alloy, its aluminized coating, and Al-Cr co-diffusion coating. Figure 1 presents the cross-sectional morphology and elemental distribution of the GH5188 alloy, aluminized coating, and Al-Cr co-diffusion coating. The GH5188 alloy matrix contained uniformly distributed spherical W-rich compounds, with other major elements evenly distributed without segregation. After aluminum diffusion using the solid powder pack method, the aluminized coating on GH5188 alloy exhibited a thickness of approximately 57 μm. The coating was relatively dense, with a uniform distribution of Al, and contained a few W-rich compounds. A distinct interface was observed between the coating and the substrate due to Al not being a primary element of the GH5188 alloy. The Al-Cr co-diffusion coating on the GH5188 alloy had a thickness of 20 μm. This coating showed the distinct layering of Al and Cr elements, with Cr enrichment near the coating-substrate interface and Al enrichment in the middle of the coating. Some W-rich compound aggregation was observed near the interface close to the substrate. The phase composition of the three samples was characterized with XRD, as shown in Figure 2. The primary phase of the GH5188 matrix was an austenitic matrix structure composed of Co-Cr-Ni, with some Si_2_W dispersed strengthening phases. The aluminized coating contained a substantial amount of Co-Al intermetallic compounds, such as CoAl, Al_5_Co_2_, and Co_4_Al_13_, with minor amounts of Al_4_Ni_3_ and CrCo_2_Al intermetallic compounds. On the surface of the Al-Cr co-diffusion coating of the GH5188 alloy, Cr_23_C_6_ and minor Al and Ni intermetallic compounds were predominantly present.

**Figure 3:**
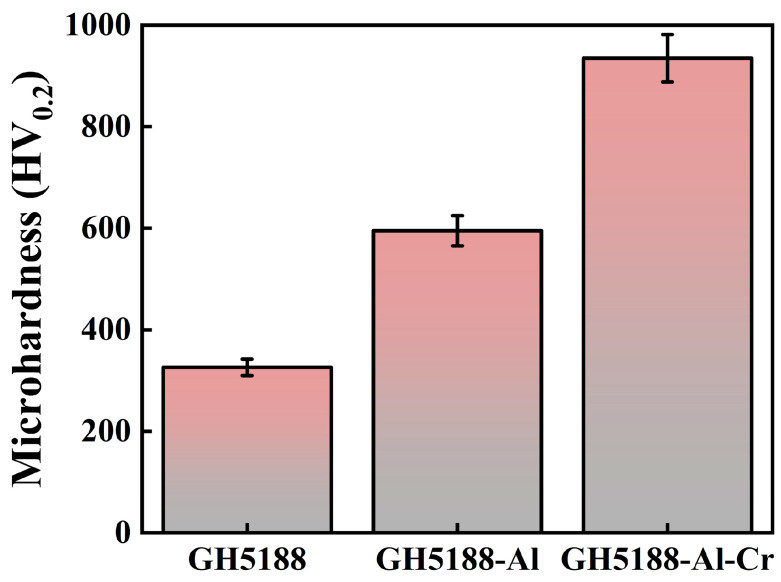
Micro Vickers hardness of the surface of the GH5188 Alloy, aluminum-diffusion coating, and Al-Cr co-diffusion coating.

The microstructure of different samples directly impacts their surface hardness, which in turn affects their tribological performance. To further analyze the impact of microstructure on hardness, the surface hardness of the GH5188 alloy, aluminized coating, and Al-Cr co-diffusion coating was compared. The results are shown in Figure 3. The hardness of the GH5188 substrate was measured at 326 HV_0.2_. The hardness of the aluminized coating was significantly higher at 595 HV_0.2_, while the Al-Cr co-diffusion coating exhibited the highest hardness at 935 HV_0.2_. Compared with the substrate, the hardness of the aluminized coating increased by approximately 82.5% and the hardness of the Al-Cr co-diffusion coating increased by approximately 186.8%.

### 3.2. Tribological Performance

**Figure 4:**
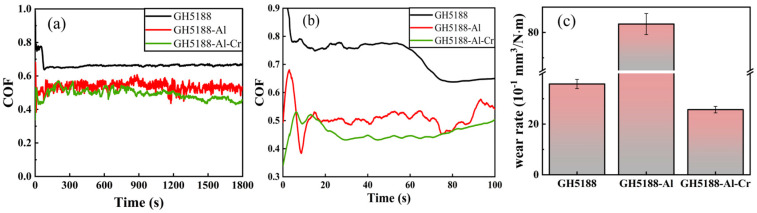
Coefficient of friction and wear rate of different samples at 700 °C: (**a**) coefficient of friction; (**b**) coefficient of friction in running-in stage; (**c**) wear rate.

During the course of this work, high-temperature friction and hardness tests were repeated three times under identical conditions for the same coating, and error bars were added to the relevant results for calibration. Figure 4 demonstrates the high-temperature friction and wear performance of the three samples at 700 °C. Considering that actual working conditions typically involve an initial running-in period, the first 100 s of the friction process were analyzed as the running-in stage, as shown in Figure 4b. After 1800 s of high-temperature friction, the friction coefficients of the three samples stabilized, as illustrated in Figure 4a. The GH5188 alloy exhibited a friction coefficient of approximately 0.65. The aluminized coating (GH5188-Al) had a friction coefficient fluctuating around 0.5, showing less stability compared with the GH5188 alloy. The Al-Cr co-diffusion coating had the lowest friction coefficient, around 0.45, and displayed a gradual decreasing trend over time. The friction coefficients of the aluminized and Al-Cr co-diffusion coatings were reduced by about 23% and 31%, respectively, compared with the substrate. In the first 100 s of the friction process, as shown in Figure 4b, the friction coefficients of all samples were generally higher and less stable. Particularly, the Al-Cr co-diffusion coating exhibited a slow decrease in the friction coefficient over time, indicating better stability during the friction process. However, evaluating the high-temperature friction performance of materials requires not only an assessment of the friction coefficient but also an examination of the wear rate. Figure 4c presents the wear rates of the three samples after 30 min of high-temperature friction at 700 °C. The aluminized coating exhibited the highest wear rate, approximately 83.314 × 10^−1^ mm^3^/N·m, significantly higher than the 36.824 × 10^−1^ mm^3^/N·m of the GH5188 alloy, with the wear rate of the aluminized coating being about 2.3 times that of the substrate. The Al-Cr co-diffusion coating had the lowest wear rate, only 25.706 × 10^−1^ mm^3^/N·m, which was 30.5% lower than that of the substrate. Details are shown in the Table 3.

**Figure 5:**
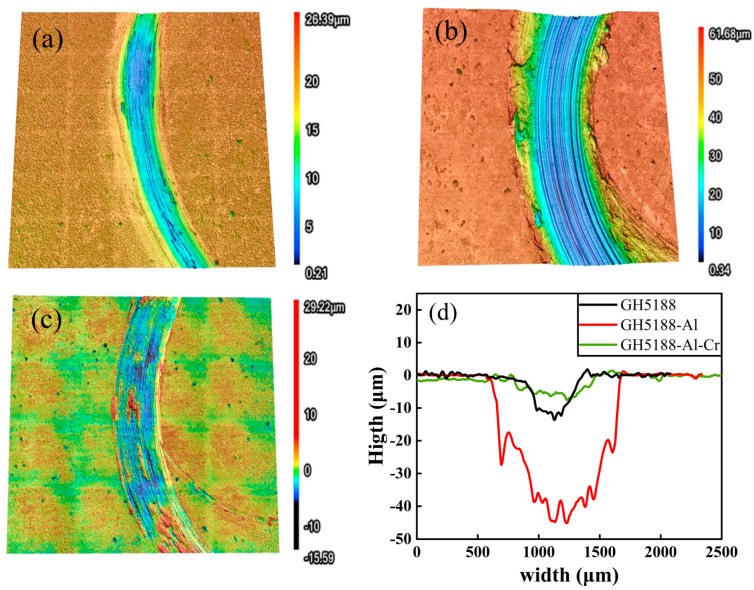
3D morphology and cross-sectional profile of wear tracks on different samples after high-temperature friction at 700 °C: (**a**) GH5188 alloy; (**b**) aluminum-diffusion coating; (**c**) Al-Cr co-diffusion coating; (**d**) cross-sectional profile of wear tracks.

A laser confocal microscope was employed to further analyze the wear conditions of the three samples after high-temperature friction. Figure 5a–d present the three-dimensional morphologies and cross-sectional profiles of the wear tracks. It was evident that the aluminized coating exhibited the greatest width and depth of wear tracks. The deepest point reached 45 μm, and the wear track width was approximately 1000 μm, characterized by a “deep and wide” morphology. Additionally, a significant accumulation of wear debris layers was observed on both sides of the wear tracks. In contrast, the wear tracks of the Al-Cr co-diffusion coating showed a smaller accumulation of wear debris and the shallowest wear depth, approximately 7 μm, which was only 15% of the depth observed in the aluminized coating, indicating superior wear resistance. The GH5188 alloy exhibited a maximum wear depth of about 11 μm and a wear track width of approximately 400 μm.

The depth and width of wear tracks are crucial indicators of a material’s wear resistance. Although both the aluminized coating and the Al-Cr co-diffusion coating showed significant improvements in friction coefficients, the wear rate analysis revealed different levels of performance. The aluminized coating, due to its lower hardness and inferior wear resistance, resulted in deeper and wider wear tracks. In contrast, the Al-Cr co-diffusion coating demonstrated excellent wear resistance, attributed to its advantageous elemental composition and microstructure. Next, the high-temperature tribological behavior and wear resistance mechanisms of the GH5188 alloy and its coatings will be discussed, focusing on the microstructure and elemental distribution of the wear tracks on the surfaces of the three samples.

## 4. Discussion

### 4.1. Study of High-Temperature Friction Behavior of GH5188 Alloy at 700 °C

**Figure 6:**
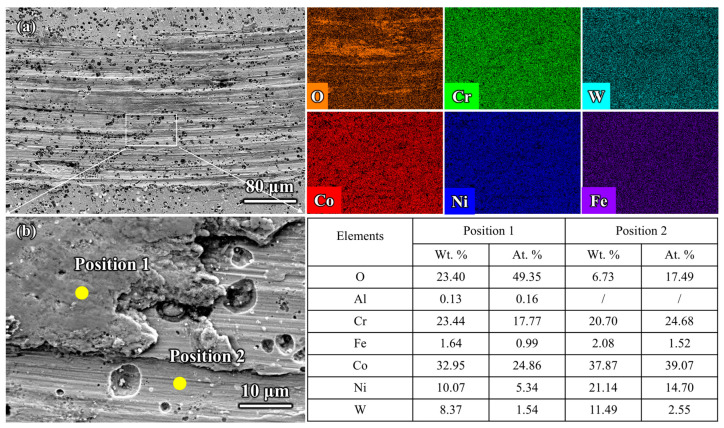
Wear track morphology and element distribution on the surface of the GH5188 alloy after high-temperature friction at 700 °C: (**a**) overall wear track morphology and element distribution; (**b**) local wear track features and semi-quantitative elemental results.

**Figure 7:**
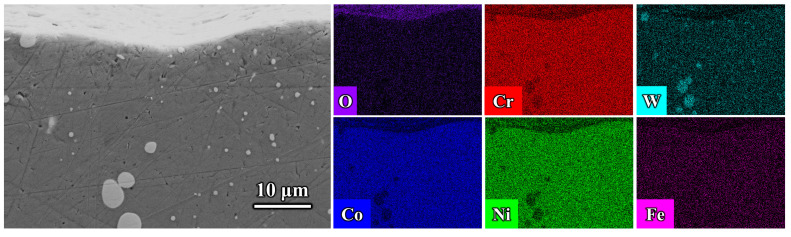
Cross-sectional morphology and element distribution of wear tracks on the GH5188 alloy after high-temperature friction at 700 °C.

The core findings of this study not only apply to cobalt-based alloys but can also be generalized to other materials and engineering contexts, providing valuable insights for the design of high-temperature coatings, the development of materials for extreme environments, and interdisciplinary applications [31,32]. Based on the surface hardness and high-temperature friction performance results of the three samples in Figure 3 and Figure 4, the aluminized coating, despite having a higher hardness than the GH5188 alloy substrate, did not exhibit significant wear resistance and instead increased the wear rate. However, when the aluminized coating was modified with Cr elements, its high-temperature wear resistance significantly improved. This indicates that the tribological performance of the samples was not directly related to surface hardness. The microstructure of the friction interface is the key factor influencing wear resistance. As the supporting substrate for the coating system, the tribological behavior of the GH5188 alloy at 700 °C directly determines the service boundary conditions of the coating system [33]. As shown in Figure 6, the alloy surface exhibited a typical plowing morphology with discontinuously distributed oxide debris. As shown in Figure 7, Pits caused by the detachment of wear debris were also present on the wear track surface. Energy dispersive spectroscopy (EDS) analysis indicated that these debris particles were rich in Co (~24.86 at.%), Cr (~17.77 at.%), and O (~49.35 at.%). Even at the debris detachment sites, the main elements remained O, Cr, and Co. During high-temperature friction, the GH5188 alloy undergoes a cyclical interaction of wear forces and thermal effects. Under contact stress, the original surface oxides are disrupted, exposing fresh metal surfaces to high-temperature oxidation. Co and Cr elements oxidize preferentially, and these oxides are ground into flaky debris under shear forces, with some embedding in the plow grooves to form a discontinuous lubricating layer. However, the observation of the wear track surface and cross-sections revealed no formation of a continuous glaze layer. This transient oxidation behavior is closely related to the low Cr solubility (~25 wt.%) in the austenitic matrix (γ-Co) of the GH5188 alloy [34]. During high-temperature friction, Cr elements cannot continuously diffuse to the surface to form a dense oxide film, resulting in characteristic localized oxide layer fracturing.

**Figure 8:**
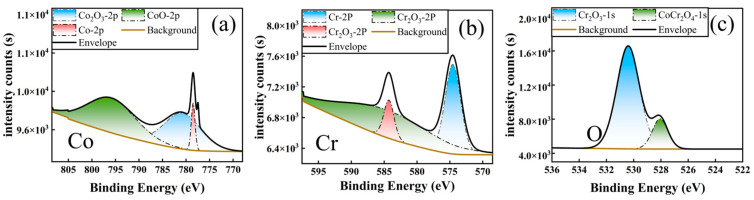
XPS Spectra of the wear tracks on the GH5188 alloy surface after high-temperature friction: (**a**) O; (**b**) Co; (**c**) Cr.

To further analyze the main components of wear debris on the GH5188 alloy, X-ray photoelectron spectroscopy (XPS) depth profiling was utilized to examine the chemical states of surface oxides (Figure 8). The Cr 2p_3/2_ peak, located at 576.8 eV, corresponded to the characteristic of Cr_2_O_3_ [35]; the Co 2p_3/2_ peak showed a significant signal at 780.5 eV, indicating the presence of a CoCr_2_O_4_ spinel structure [36]. It is noteworthy that Cr_2_O_3_ possesses a high hardness (HV 2300) and a low oxygen diffusion coefficient (1.2 × 10^−14^ m^2^/s at 700 °C) [37], which can effectively suppress the plastic deformation of the subsurface of a wear track. Meanwhile, the CoCr_2_O_4_ spinel structure, due to its lamellar shear properties, provides solid lubrication at the friction interface [38]. This composite structure, with the synergistic effect of the hard substrate (Cr_2_O_3_) and the lubricating phase (CoCr_2_O_4_), plays a crucial role in enhancing the high-temperature friction performance of the GH5188 alloy.

However, the protective effect of this discontinuous oxide layer is limited by structural defects. As shown in Figure 6, noticeable spalling pits appeared at the edges of the wear track. Energy dispersive spectroscopy (EDS) indicated a sharp drop in oxygen content to 17.49 at.% in this region. This may have been due to the mismatch between the oxide layer and the substrate induced by cyclic thermal stress. When local stress exceeds the interfacial bonding strength between the Cr_2_O_3_ layer and the alloy substrate [39,40,41], the oxide layer spalls off, accelerating abrasive wear.

In summary, during the high-temperature friction process of the GH5188 alloy, an initial dynamic oxidation process occurs. As the friction continues, the intermediate oxidation layer locally fractures, leading to abrasive wear. In the later stages, insufficient oxides fail to compensate for wear track defects, and spalling cycles result in cumulative damage. By preparing high-oxygen-affinity coatings on the surface of the GH5188 alloy and forming a continuous and dense oxide barrier layer, it is expected that the high-temperature friction and wear performance of the alloy substrate can be significantly improved.

### 4.2. High-Temperature Friction Behavior and Oxide Debris-Shedding Mechanisms of Aluminum-Diffusion Coatings

Existing research indicates that aluminized coatings exhibit good high-temperature adhesion resistance but lack sufficient high-temperature friction performance. The Cr element, however, easily forms dense and chemically stable oxides in high-temperature environments, directly enhancing an alloy’s high-temperature wear resistance [42]. Theoretically, Cr-modified aluminized coatings can achieve integrated high-temperature adhesion and wear resistance [43]. This section details the high-temperature friction behavior of aluminized coatings and the high-temperature wear mechanisms after Cr modification.

**Figure 9:**
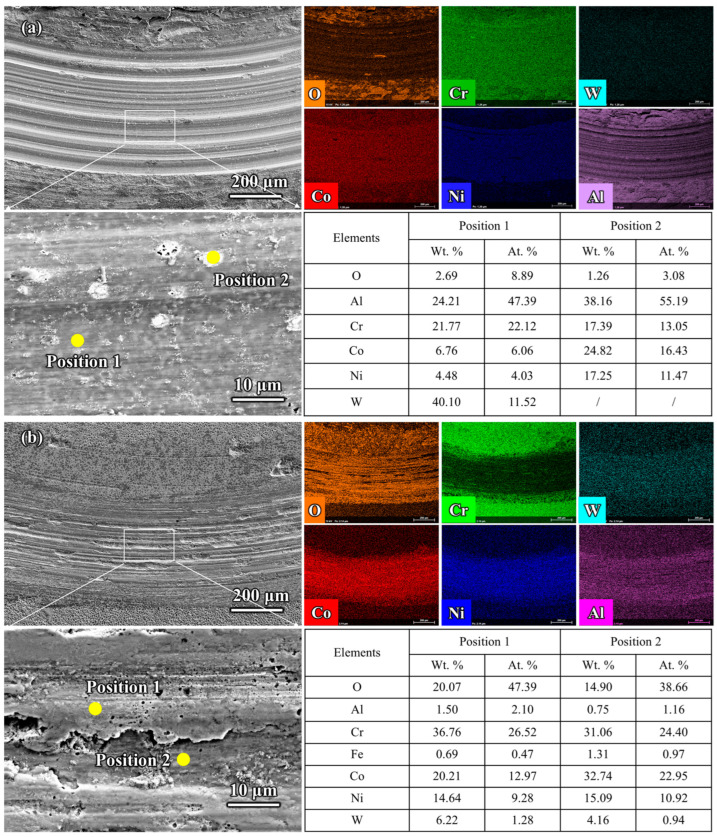
Comparative results of wear track morphology and element distribution on the surface of aluminum-diffusion coating and Al-Cr co-diffusion coating after high-temperature wear: (**a**) aluminum-diffusion coating; (**b**) Al-Cr co-diffusion coating.

Figure 9a presents the comparative results of surface wear track morphology and elemental distribution after high-temperature wear for the aluminized coatings and Al-Cr co-deposited coatings. The results indicate that the wear tracks on the aluminized coatings exhibited typical brittle wear characteristics, with evident plowing morphology, and a maximum wear depth reaching up to 40 μm. No significant oxide wear debris was observed on the wear tracks, and the oxygen content on the wear track surface was only 8.89 at.%. Additionally, numerous high-contrast circular impurities were detected on the wear tracks, mainly composed of Al, Cr, Co, Ni, and O elements.

Figure 9b shows the wear track morphology and elemental distribution of Al-Cr co-deposited coatings after high-temperature friction at 700 °C. Compared with the wear tracks on the aluminized coating and the GH5188 alloy, it is evident that the wear tracks of the Al-Cr co-deposited coatings contained a substantial amount of oxide debris. Most of these oxides were smooth and continuously distributed on the wear tracks, with some oxide debris spallation pits and plowing morphology also present. The oxide debris mainly consisted of O (approximately 47.39 at.%), Cr (approximately 26.52 at.%), and Co (12.97 at.%). Despite the presence of spallation pits, the oxygen content at these locations (approximately 38.66 at.%) was still higher than that in the spallation pits of the wear tracks on the GH5188 alloy and its aluminized coating.

**Figure 10:**
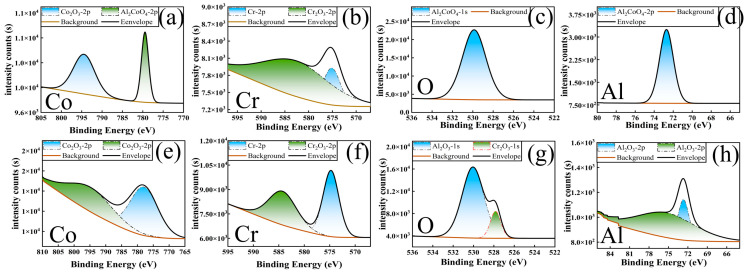
XPS spectra of wear tracks on aluminum-diffusion coating and Al-Cr co-diffusion coating after high-temperature friction at 700 °C: (**a**–**d**) aluminum-diffusion coating; (**e**–**h**) Al-Cr Co-diffusion coating.

The further analysis of the oxide species in the wear debris was conducted using XPS, and the results are shown in Figure 10. The oxides on the wear track surfaces of aluminized coatings were primarily CoAl_2_O_4_ (Co 2p_3_/_2_ = 781.2 eV, Al 2p = 74.5 eV) [44], while the oxides on the wear tracks of Al-Cr co-deposited coatings were mainly Cr_2_O_3_ (Cr 2p_3_/_2_ = 576.6 eV), CoCr_2_O_4_ (Co 2p_3_/_2_ = 780.8 eV), with a small amount of CoO (Co 2p_3_/_2_ = 779.5 eV) [45]. The primary oxides in the wear debris of the three samples were spinel structures. The differences in high-temperature tribological performance are fundamentally due to the stability and physicochemical properties of different oxide types in high-temperature environments.

The ionic potential (Φ) is a parameter that describes a cation’s polarizing ability and the stability of oxides. It is defined as the ratio of the cation’s charge number (Z) to its ionic radius (r):

(2) $$\Phi =\frac{Z}{r}$$

Here, Z denotes the cation’s charge number and r represents the cation’s ionic radius. The greater the ionic potential difference between two oxides, the more likely it is to form composite oxides with low melting points, low hardness, and low shear strength [46,47,48]. The oxides on the high-temperature wear surfaces of the GH5188 alloy, aluminized coatings, and Al-Cr co-deposited coatings are generally formed through the following chemical reactions:

Cr_2_O_3_(s) + CoO(s)→CoCr_2_O_4_ (3)

Al_2_O_3_(s) + CoO(s)→CoAl_2_O_4_ (4)

Based on calculations, the ionic potential of Al_2_O_3_ is 5.6 Å^−1^, that of CoO is 3.0 Å^−1^, and that of Cr_2_O_3_ is 4.9 Å^−1^. The ionic potential difference between Al_2_O_3_ and CoO is greater than that between Cr_2_O_3_ and CoO. Furthermore, there is a significant difference in the bonding characteristics of Al^3+^, Cr^3+^, and O^2−^. The Al-O bond is primarily ionic (Pauling electronegativity difference Δχ = 1.83), whereas the Cr-O bond exhibits a stronger covalency (Δχ = 1.43). This causes Cr^3+^ to more easily form stable octahedral sites in the spinel structure [49]. Additionally, CoAl_2_O_4_ in the wear tracks of aluminized coatings undergoes a θ→α-Al_2_O_3_ phase transition at 700 °C, accompanied by a 14% volume shrinkage [50], leading to tensile stress within the film (σ ≈ 500 MPa). In contrast, CoCr_2_O_4_ maintains a single-phase spinel structure at the same temperature. The higher ionic potential of Cr^3+^ inhibits oxygen ion migration (D_Cr_2_O_3__ = 1.2 × 10^−14^ m^2^/s vs. D_Al_2_O_3__ = 8.3 × 10^−14^ m^2^/s), delaying phase transition-induced structural instability. First-principles calculations show that the phase transition barrier for CoCr_2_O_4_ (ΔG* = 0.85 eV) is significantly higher than that for CoAl_2_O_4_ (ΔG* = 0.62 eV), confirming its superior thermodynamic stability [51].

Furthermore, the growth stress of oxides and the diffusion rates of elements determine the integrity and self-healing capability of wear debris on a wear surface, significantly impacting a material’s high-temperature wear resistance. The growth stress of oxides (σ_ox_) refers to the mechanical stress generated internally within an oxide during its formation or growth. This stress arises due to volume changes, structural differences, or external conditions such as temperature and pressure. The expression for oxide growth stress can be described using the modified Pilling–Bedworth model as follows:

(5) $$\sigma_{ox}=E_{ox}\cdot (PBR-1)/(3(1-\nu_{ox}))$$

Here, Eox represents the elastic modulus of the oxide (GPa), PBR (Pilling–Bedworth Ratio) is the volume ratio of the oxide to the original metal, and νox denotes the Poisson’s ratio of the oxide [52]. Based on relevant data from researchers in the field, the key parameters for oxides such as Cr_2_O_3_, CoCr_2_O_4_, and CoAl_2_O_4_ in this study are shown in the Table 4:

Based on the above results, it is evident that the growth stress of CoAl_2_O_4_ is the highest, followed by CoCr_2_O_4_, and the lowest is Cr_2_O_3_. During high-temperature friction, CoAl_2_O_4_ oxide debris is most prone to spalling under the applied wear forces. Simultaneously, under the high-temperature conditions of the friction process, Al and Cr elements are easily oxidized and form oxides anew, contributing to the wear debris. According to Wagner’s oxidation theory, the diffusion activation energy of Al^3+^ in CoAl_2_O_4_ (Q_Al_ = 168 kJ/mol) is higher than that of Cr^3+^ in CoCr_2_O_4_ (Q_Cr_ = 145 kJ/mol), resulting in a lower diffusion rate for Al^3+^ (D_Al_ = 2.1 × 10^−16^ m^2^/s vs. D_Cr_ = 5.0 × 10^−16^ m^2^/s, 700 °C). The slower diffusion of Al^3+^ hinders the timely repair of local defects in the CoAl_2_O_4_ oxide film, whereas the high mobility of Cr^3+^ facilitates the dynamic healing of oxides such as CoCr_2_O_4_ and Cr_2_O_3_. Chromium-rich oxides possess strong bonding capabilities, making them prone to form protective tribolayer films that enhance oxidation resistance and reduce wear. This Cr-modified oxide design can be applied to other alloys requiring high-temperature friction resistance [53].

**Figure 11:**
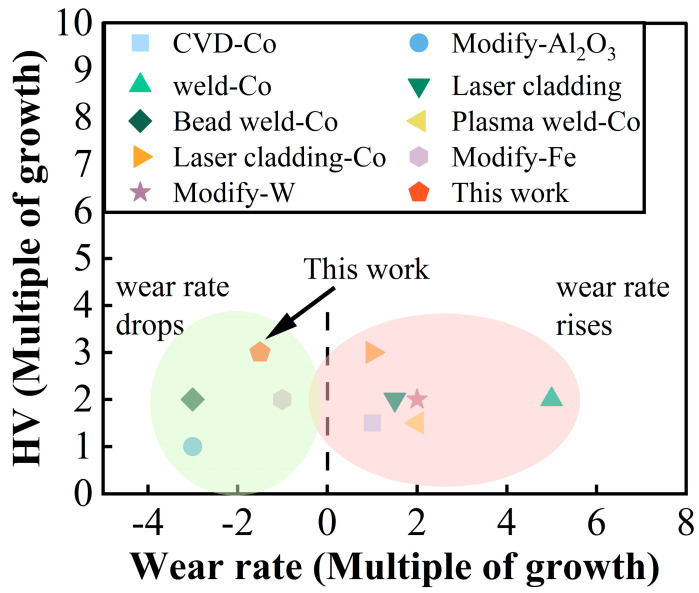
Comparison of the high-temperature friction performance of the Al-Cr co-diffusion coating in this study with various coatings reported in the literature.

The metal co-diffusion coating prepared using the powder embedding method in this study not only significantly enhanced the hardness of the cobalt-based alloy but also effectively improved its high-temperature friction performance. This preparation method is simple and holds substantial application potential. Figure 11 presents a comparison of the hardness and wear rates of protective coatings prepared with different methods. It can be observed that although processes such as laser cladding and overlay welding significantly increase the hardness of coatings, they also lead to higher wear rates. Conversely, methods involving the addition of oxides or metal atoms during melting can reduce wear rates but do not significantly enhance the mechanical properties of the substrate [54]. Comprehensive analysis indicates that the metal co-diffusion coating prepared using the powder embedding method in this study offers notable advantages in improving hardness and high-temperature friction performance, providing better overall performance compared with other methods. Details are shown in the Table 5. Therefore, this method holds promising potential for significantly enhancing the performance of cobalt-based alloys under high-temperature friction conditions.

## 5. Conclusions

Future research on improving the high-temperature friction performance of cobalt-based alloys should focus on material design, surface modification, lubrication strategies, and multiscale simulations. For example, molecular dynamics and finite element analysis could be used to predict the performance evolution of cobalt-based alloys under high-temperature friction. Breakthroughs in high-temperature friction performance can be achieved through interdisciplinary collaboration and technological innovation. The chromium–aluminum co-diffusion coating preparation techniques developed in this work, through the synergistic optimization of materials, processes, and environments, not only provide a methodological foundation for the development of high-temperature coatings but also promote the sustainable application of cobalt-based alloys in aerospace and other industries. This study addresses the critical need for the wear resistance of stator bushings made of GH5188 cobalt-based alloys in high-temperature service environments. The research focused on the anti-high-temperature wear mechanism of an Al-coated GH5188 alloy modified with Cr via thermal diffusion technology. The main conclusions are as follows:

(1) The primary microstructure of the GH5188 alloy is austenitic, containing a small amount of Si_2_W. The main phases of the Al-coated layer are intermetallic compounds such as CrCo_2_Al and Al_5_Co_2_, while the primary phases on the surface of the Al-Cr co-diffusion layer are Cr_23_C_6_ and AlNi. The hardness of the GH5188 substrate is 326 HV_0.2_, the Al-coated layer reaches 595 HV_0.2_, and the Al-Cr co-diffusion layer achieves a hardness of 935 HV_0.2_. Compared with the substrate, the hardness of the Al-coated layer increased by approximately 82.5% and the hardness of the Al-Cr co-diffusion layer increased by approximately 186.8%. The high-temperature friction performance of the three samples was directly related to the structure and content of oxide debris on the wear scar surfaces. The results indicate that after high-temperature friction at 700 °C, the Al-coated layer exhibited the highest wear volume and the lowest content of oxide debris on the wear scar surface, primarily composed of CoAl_2_O_4_. The Al-Cr co-diffusion layer demonstrated the least wear, followed by the GH5188 alloy. The wear debris of both the Al-Cr co-diffusion layer and the GH5188 alloy consisted of CoCr_2_O_4_ and Cr_2_O_3_, with the Al-Cr co-diffusion layer having the highest debris content.
(2) The high-temperature wear resistance of oxides on the wear scar surface is associated with the thermodynamic stability, high-temperature growth stress, and high-temperature oxidation of the wear debris. The CoAl_2_O_4_ debris of the Al-coated layer exhibits the highest growth stress, and the diffusion rate of Al^3+^ at high temperatures is lower than that of Cr^3+^, making it difficult to form stable, lubricating oxides after debris spalling. Conversely, the Cr-modified Al-coated layer increases the Cr content on the alloy surface, forming CoCr_2_O_4_ and Cr_2_O_3_ with lower growth stress. The higher diffusion rate of Cr^3+^ at high temperatures aids in the formation of lubricating wear debris.

## Figures and Tables

**Table 1:** Chemical composition of the base material (mass%).

Elements	Co	Ni	Cr	W	Fe	Mn
mass/%	remain	22.00	22.00	15.00	3.00	1.25
elements	Si	Cu	B	C	P	S
mass/%	0.40	0.070	0.15	0.10	0.020	0.015

**Table 2:** Chemical composition of diffusion agents (wt.%).

Coating	Diffusion Agent Composition				Temperature	Time
	Al	Al_2_O_3_	Cr_2_O_3_	NH_4_I		
Al	30	65		5	900 °C	6 h
Al-Cr	10	65	20	5	1090 °C	6 h

**Table 3:** Tribological test results of different samples.

	GH5188	GH5188-Al	GH5188-Al-Cr
Average COF	0.65	0.5	0.45
Wear rate/×10^−1^ mm^3^/N·m	36.824	83.314	25.706
Wear scar depth/μm	11	45	7
Wear scar width/μm	400	1000	650

**Table 4:** Parameters and results of oxide growth stress calculation for three types of oxides.

Types of Oxides	PBR	E_ox_	$\nu_{ox}$	$\sigma_{ox}$ (GPa)
Cr_2_O_3_	1.03	280	0.30	4
CoCr_2_O_4_	1.65	240	0.28	72.2
CoAl_2_O_4_	1.99	200	0.25	88

**Table 5:** Comparison of the results for comprehensive performance enhancement.

	CVD-Co	Modify-Al_2_O_3_	Weld-Co	Laser Cladding	Weld-Co
Hardness improvement	1.5	1	2	2	2
Wear resistance improvement	−1	3	−5	−1.5	3
	**Plasma Weld-Co**	**Laser Cladding-Co**	**Modify-Fe**	**Modify-W**	**This Work**
Hardness improvement	1.5	3	2	2	3
Wear resistance improvement	−2	−1	1	−2	1.5

## Data Availability

The original contributions presented in this study are included in the article. Further inquiries can be directed to the corresponding authors.
